# Single‐cell analysis reveals metastatic cell heterogeneity in clear cell renal cell carcinoma

**DOI:** 10.1111/jcmm.16479

**Published:** 2021-03-23

**Authors:** Kun Liu, Rui Gao, Hao Wu, Zhe Wang, Guang Han

**Affiliations:** ^1^ Department of Anesthesiology Shengjing Hospital of China Medical University Shenyang China; ^2^ Department of Gastrointestinal Oncology Cancer Hospital of China Medical University Liaoning Cancer Hospital & Institute Shenyang China

**Keywords:** epithelial‐mesenchymal transition, intratumour heterogeneity, metastatic renal cell carcinoma, single‐cell analysis, tumour microenvironment

## Abstract

Renal cell carcinoma (RCC) is one of the leading causes of cancer‐related death worldwide. Tumour metastasis and heterogeneity lead to poor survival outcomes and drug resistance in patients with metastatic RCC (mRCC). In this study, we aimed to assess intratumoural heterogeneity (ITH) in mRCC cells by performing a combined analysis of bulk data and single‐cell RNA‐sequencing data, and develop novel biomarkers for prognosis prediction on the basis of the potential molecular mechanisms underlying tumorigenesis. Eligible single‐cell cohorts related to mRCC were acquired using the Gene Expression Omnibus (GEO) dataset to identify potential mRCC subpopulations. We then performed gene set variation analysis to understand the differential function in primary RCC and mRCC samples. Subsequently, we applied weighted correlation network analysis to identify coexpressing gene modules that were related to the external trait of metastasis. Protein‐protein interactions were used to screen hub subpopulation‐difference (sub‐dif) markers (ACTG1, IL6, CASP3, ACTB and RAP1B) that might be involved in the regulation of RCC metastasis and progression. Cox regression analysis revealed that *ACTG1* was a protective factor (HR < 1), whereas the other four genes (*IL6*, *CASP3*, *ACTB* and *RAP1B*) were risk factors (HR > 1). Kaplan‐Meier survival analysis suggested the potential prognostic value of these sub‐dif markers. The expression of sub‐dif markers in mRCC was further evaluated in clinical samples by immunohistochemistry (IHC). Additionally, the genetic features of sub‐dif marker expression patterns, such as genetic variation profiles, correlations with tumour‐infiltrating lymphocytes (TILs), and targeted signalling pathway activities, were assessed in bulk RNA‐seq datasets. In conclusion, we established novel subpopulation markers as key prognostic factors affecting EMT‐related signalling pathway activation in mRCC, which could facilitate the implementation of a treatment for mRCC patients.

## 1 INTRODUCTION

Renal cell carcinoma (RCC) is characterized by various genetic abnormalities, and the accompanying clinical and biological heterogeneity plays a crucial role in the modification of drug resistance, signalling networks, distant metastasis and prognosis.^1^, ^2^, ^3^ Clear cell RCC (ccRCC) is the most prevalent histopathological type, constituting more than 85% of metastatic RCC (mRCC) cases and showing a poor prognosis. Nearly 20% of ccRCC cases present with de novo metastatic disease at initial diagnosis, and the 5‐year overall survival (OS) rate of metastatic cases is as low as 10%.^4^, ^5^

Over the last 12 years, the clinical management of mRCC has progressed significantly. Multiple targeted agents have been developed to block the activity of known tyrosine kinases and signalling pathways, such as inhibitors of the mammalian target of rapamycin (mTOR), platelet‐derived growth factor (PDGF) and vascular endothelial growth factor (VEGF) pathways, which mediate crucial network functions involved in ccRCC, metastasis and angiogenesis.^6^, ^7^, ^8^, ^9^, ^10^ Although targeted drugs for VEGF or PI3K/mTOR are effective first‐line treatment options, many patients with mRCC eventually tend to develop drug resistance. The median time of disease progression into a drug‐resistant phenotype is approximately 6‐15 months, depending on the therapeutic programmes and intratumoural heterogeneity (ITH).^11^, ^12^

Epithelial‐to‐mesenchymal transition (EMT), a process in which epithelial cells lose their apical‐basal polarity and concomitantly acquire a migratory phenotype,^13^, ^14^ is a key step in tumour metastasis. The EMT programme in tumour metastasis adapts to the ITH and constantly evolving microenvironment to allow tumour cells to successfully metastasize.^13^ Since EMT is dynamically regulated during metastasis, more studies on the molecular regulators of EMT could shed light on the therapeutic approaches to inhibit tumour colonization. However, the mechanisms by which EMT heterogeneity cooperates with the tumour microenvironment (TME) and activates distinct downstream signalling pathways in metastatic sites to promote tumour progression remains unclear.

The emerging single‐cell RNA‐sequencing (scRNA‐seq) technology provides deeper insights into transcriptome expression profiles at a single‐cell resolution and a detailed understanding of ITH.^15^, ^16^, ^17^, ^18^ Tumour metastasis is an evolutionary process, and cells may acquire novel or different phenotypes through selection.^19^, ^20^ scRNA‐seq has helped reveal unidentical subpopulations, greatly facilitating the development of novel approaches for improving precision targeted therapy.^21^ Nevertheless, elucidation of the role of ITH in mRCC cells and its contribution to drug resistance remains a challenge, and this information is expected to have profound implications in improving our understanding of the process of tumour subclonal evolution and development of effective treatment regimens. Thus, in this study, we sought to assess mRCC cell heterogeneity by analysing a combination of bulk data and scRNA‐seq data.

We hypothesized that alterations in gene expression profiles during the evolutionary process of tumour metastasis affect the phenotype of metastatic cancer cells, resulting in the activation of distinct signalling pathways and drug resistance to specific treatments. Our analyses indicated that EMT activation reflects a main cell subset with distinct mechanisms of action in mRCC. Using publicly available human sequencing datasets (The Cancer Genome Atlas [TCGA] and Gene Expression Omnibus [GEO] Series [GSE73121]) and immunohistochemistry (IHC) analysis, we confirmed that IL6, CASP3, ACTB, ACTG1 and RAP1B, which are referred to as subpopulation‐difference or sub‐diff markers, are co‐regulators of the differentially expressed gene (DEG) network between two metastatic subpopulations and play key roles in RCC metastasis. We further explored the expression patterns and genetic mutations of these five sub‐dif markers to evaluate their influence on the prognosis of RCC in large patient cohorts. These results indicated that the activation of pathways differed in mRCC subpopulations and that EMT was the main pathway. Sub‐dif markers are key prognostic factors that participate in EMT induction in mRCC subpopulations. Furthermore, the correlations of sub‐dif markers with immune infiltration and drug response were analysed to ascertain their value as molecular markers for predicting a subpopulation evolution course, and as novel potential pharmacological targets.

## 2 METHODS

### 2.1 Gene expression data and IHC‐confirmed patient population

Bulk RNA‐seq data for kidney renal clear cell carcinoma (KIRC) tumours and normal samples, including level‐3 RNA‐seq data and clinical data, were downloaded from the TCGA data portal (https://gdc.nci.nih.gov) and used as bulk RNA‐seq data to explore sub‐dif marker gene function.

Using the single‐cell sequencing data of 118 cell samples (after removing three bulk RNA‐seq samples from the 121 cell samples) from patients with primary RCC and mRCC, single‐cell transcriptome profiles were obtained from the GEO repository under the accession number GSE73121. The criteria for filtering single cells for downstream analyses included exclusion of low‐quality cells (<200 genes/cell, <3 cells/gene and >10% mitochondrial genes) and log2‐transformation of gene expression levels by using the ScaleData R function, as described in the Seurat manual. Subsequent data analysis was performed using R software (version 4.0.2) and the Seurat package (version 2.3.4).

For the validation study, we selected 30 pairs of RCC tissues, matched non‐tumorous adjacent tissues and lung metastatic tissues from patients with a definite histological diagnosis at the Liaoning Province Cancer Hospital & Institute between 2015 and 2019. The study protocol was approved by the Ethics Committee of Liaoning Province Cancer Hospital & Institute (Ethical Approval Number: 20201252).

### 2.2 Gene set variation analysis of primary and metastatic tumour subpopulations

To estimate the differential activities of 50 hallmark pathway gene signatures between primary ccRCC and metastatic ccRCC, we implemented the gene set variation analysis (GSVA) algorithm in the scRNA‐seq data. Significantly enriched pathways were identified based on a |logFoldChange| ≥ 1 and an adjusted *P* value of <0.05. In addition, to evaluate whether the metastatic subpopulations were highly enriched with EMT‐ and VEGF‐activated pathways, GSVA was employed to determine the pathway gene set activity score for each sample by using a non‐parametric and unsupervised algorithm in the GSVA R package.^22^

### 2.3 Principal component analysis and t‐distributed stochastic neighbour embedding analyses in metastasis subgroups

Based on the assumption that genes capturing cell heterogeneity often display high variability, we focused on identifying highly variable genes (HVGs) by using the FindVariableFeatures function and used them for subsequent analyses.^23^ Principal component analysis (PCA) was conducted on the scaled HVGs, which returned six principal components (PCs) based on Cattell's screen test, and the first four principal components were chosen for visualization (Figure S1). The six returned PCs were presented for t‐distributed stochastic neighbour embedding (tSNE) dimension reduction to obtain a two‐dimensional representation of the cell state. The FindClusters function was used for clustering to realize a modular and optimized clustering algorithm of the shared nearest neighbour, which resulted in the formation of two clusters, and a resolution of 2.2 was selected. We further analysed the gene expression patterns of the mRCC subpopulations and identified 734 significant DEGs with dynamic expression changes under the threshold of an adjusted *P* value of <0.05. Cluster‐specific marker genes from the two clusters were identified and presented as violin plots and tSNE plots.

### 2.4 Defining core subpopulation differential genes in metastatic cancer cells

Weighted correlation network analysis (WGCNA) was used to acquire metastatic subpopulation‐related protein clusters.^24^ To identify the gene co‐expression modules, a hierarchical clustering analysis and a soft threshold power were assigned to group genes with similar expression patterns, and co‐expression networks were created. The networks consisted of highly similar co‐expression modules, and the eigengenes of these modules were further determined. Finally, Pearson correlations between the module eigengenes and clinical data were displayed. A protein‐protein interaction (PPI) network was constructed using the STRING online portal (https://string‐db.org/). A protein with a contribution >6 was selected to construct the PPI network, which was visualized using Cytoscape software (version 3.6.1) (http://www.cytoscape.org).^25^

### 2.5 Analysis of sub‐dif marker expression patterns and prediction of drug sensitivity

The cBioPortal database (http://www.cbioportal.org) serves as a web resource for exploration, visualization and analysis of multidimensional cancer genomics data, and it contains DNA copy number, DNA methylation, transcriptome, micro RNA and non‐synonymous mutation data.^26^, ^27^ In this study, the cBioPortal database was used for systematic analysis of the single‐nucleotide variation profile of the sub‐dif markers. Gene set cancer analysis (GSCALite), a web‐based platform for gene set cancer analysis,^28^ was applied to analyse the drug sensitivity of the sub‐dif marker genes along with the pathway activities of TCGA‐KIRC samples. We further evaluated the correlation between gene expression and IC50 using the Genomics of Drug Sensitivity in Cancer (GDSC) database.

### 2.6 Tumour IMmune Estimation Resource database analysis

The Tumor IMmune Estimation Resource (https://cistrome.shinyapps.io/timer/) is a web tool that provides a robust and comprehensive estimation of immune infiltration levels in diverse cancer types.^29^ Correlation analysis of sub‐dif marker expression with tumour‐infiltrating immune cells (TIICs) was conducted using the ‘Gene’ module. Prognostic analysis of sub‐dif marker expression and TIICs was conducted using the ‘Cox’ module. The *x*‐axis of the scatterplot represents the expression level of TIICs, whereas the *y*‐axis represents the expression level of the sub‐dif markers. All gene expression levels were represented by log2 RNA‐Seq by Expectation‐Maximization (RSEM), and the adjusted *P* value of <0.05 was considered to be statistically significant.

### 2.7 Immunohistochemistry

Tissue microarrays (TMAs) containing samples from 20 metastatic renal carcinoma patients with a definite pathological diagnosis of ccRCC were constructed for immunohistochemical analysis, as previously described.^30^ After dewaxing in xylene, rehydration in alcohol, antigen repair (0.01 M citrate buffer, pH 6.0), blocking endogenous peroxidase activity and closed with goat serum, the TMAs were incubated overnight at 4°C with rabbit anti‐human monoclonal antibodies against IL6 (dilution 1:800, clone BP53‐1; Biogenex), CASP3 (dilution 1:100, clone G168‐728; Pharmingen), ACTB (dilution 1:125, cloneFE11; Calbiochem, Oncogene Research Products), ACTG1 (dilution 1:125, cloneFE11; Calbiochem, Oncogene Research Products), and RAP1B (dilution 1:125, cloneFE11; Calbiochem, Oncogene Research Products). The TMAs were then incubated at room temperature with secondary antibodies (ab97080, goat anti‐rabbit, 1:2000; ab97040, goat anti‐mouse, 1:500; Abcam) for 20 minutes and horseradish peroxidase‐labelled Streptase ovalbumin for 20 minutes, and DAB was used for colour development and counterstained with haematoxylin. IHC staining was scored independently by two expert pathologists to determine the degree of staining positivity in each sample. The percentage of the positively stained area was 0 (fewer than 10% positive cells), 1 (10%‐25% positive cells), 2 (26%‐50% positive cells) and 3 (50% or more positive cells). The final IHC score was defined as 0 (−, negative expression), 1‐2 (+, weak expression), 3‐4 (++, medium expression) and 5‐6 (+++, strong expression).

## 3 RESULTS

### 3.1 Data availability and processing

**FIGURE 1:**
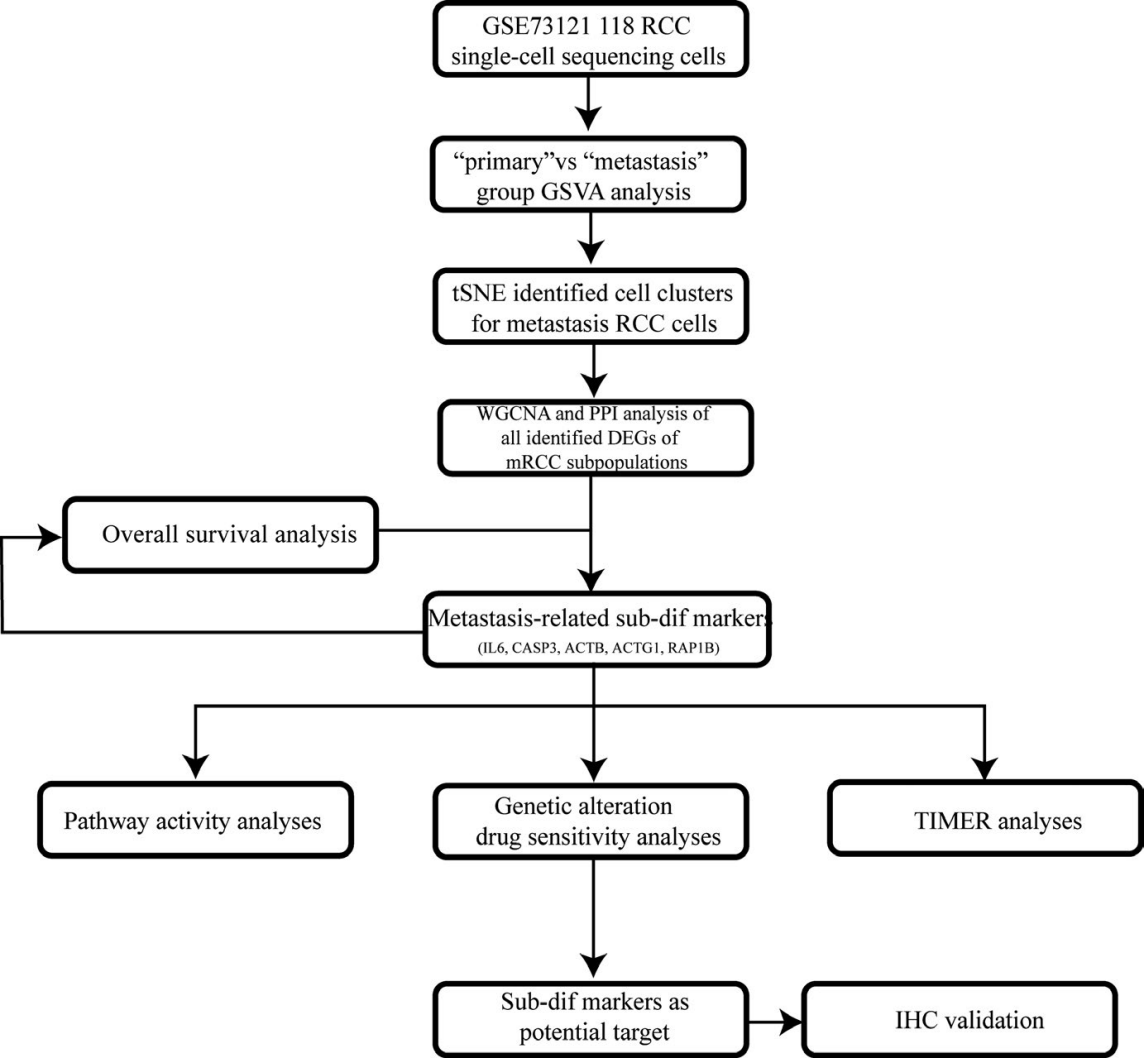
Flow diagram of the study protocol

**FIGURE 2:**
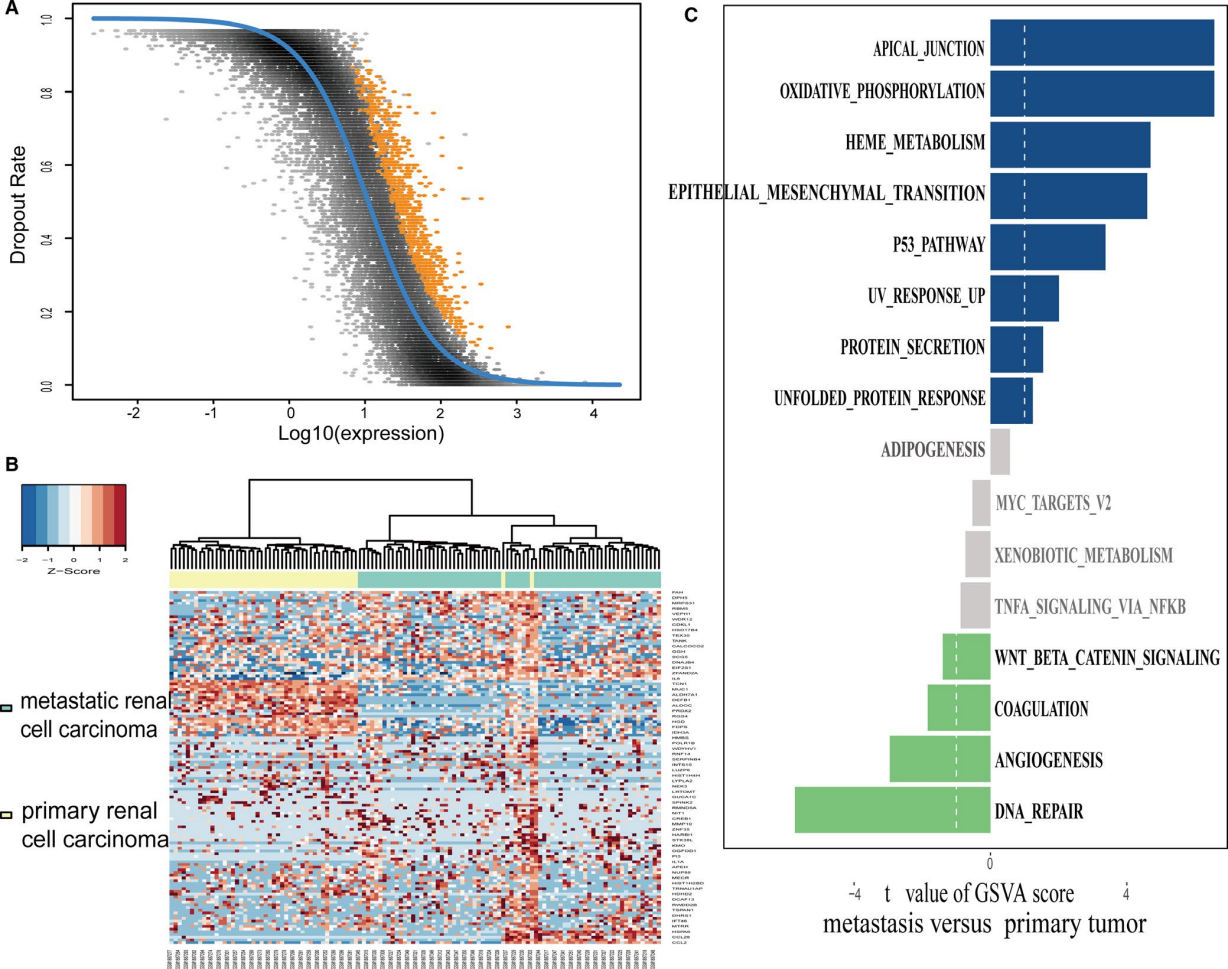
Differential distribution patterns and involved pathways between primary RCC and metastatic RCC. A, A total of 3719 high‐variant genes were identified through the M3Drop R package, and the yellow dots represent the genes with a high dropout rate. Fitting analysis suggests that these genes may be differentially expressed in cell subpopulations. B, Clustering heatmap generated by unsupervised hierarchic clustering reveals that the high‐variant genes discriminate primary RCC from metastatic RCC. C, Differences in pathway activities scored per cell using GSVA between tumour cells isolated from primary RCC or metastatic RCC. *t* values are independent of effects from the patient of origin

Figure 1 shows the flow diagram for study enrolment. After applying quality control and filtering for cells and genes, we retained 12 135 genes and 118 cells from patient‐derived primary RCC and mRCC; of these, a total of 3719 HVGs were identified using the M3Drop R package for downstream analysis (Figure 2A). The top 200 HVGs were selected for unsupervised cluster analysis, and the results (Figure 2B) showed two significantly different distribution patterns between the primary RCC tissue and mRCC samples, indicating differential expression patterns between the primary and metastatic sites in RCC. Functional differences between the primary and metastatic cancers were further explored using GSVA (Figure 2C) because one study showed that the two tumour cell clusters exhibit differences in the hallmark pathway gene signatures.^31^ The direct comparison revealed apical_junction, oxidative_phosphorylation, EMT, and P53_pathway as the top four enriched signatures in mRCC. Comparatively, angiogenesis and DNA repair were the enriched signatures in the primary RCC. Indeed, a study indicated that cell‐cell adhesion and baso‐apical polarity imply the loss of epithelial properties resulting from EMT.^32^ Angiogenesis and remarkably changed metabolic pathways have been shown to be instrumental for cancer cell metastasis.^33^, ^34^ Taken together, these findings indicate that tumour metastatic cells are remodelled to up‐regulate their EMT, angiogenesis and inflammatory pathway activities. These data extend the findings of other studies that have demonstrated a synergistic effect of tumour vessel normalization and EMT on tumour metastasis.

### 3.2 Single‐cell expression atlas of RCC metastases

**FIGURE 3:**
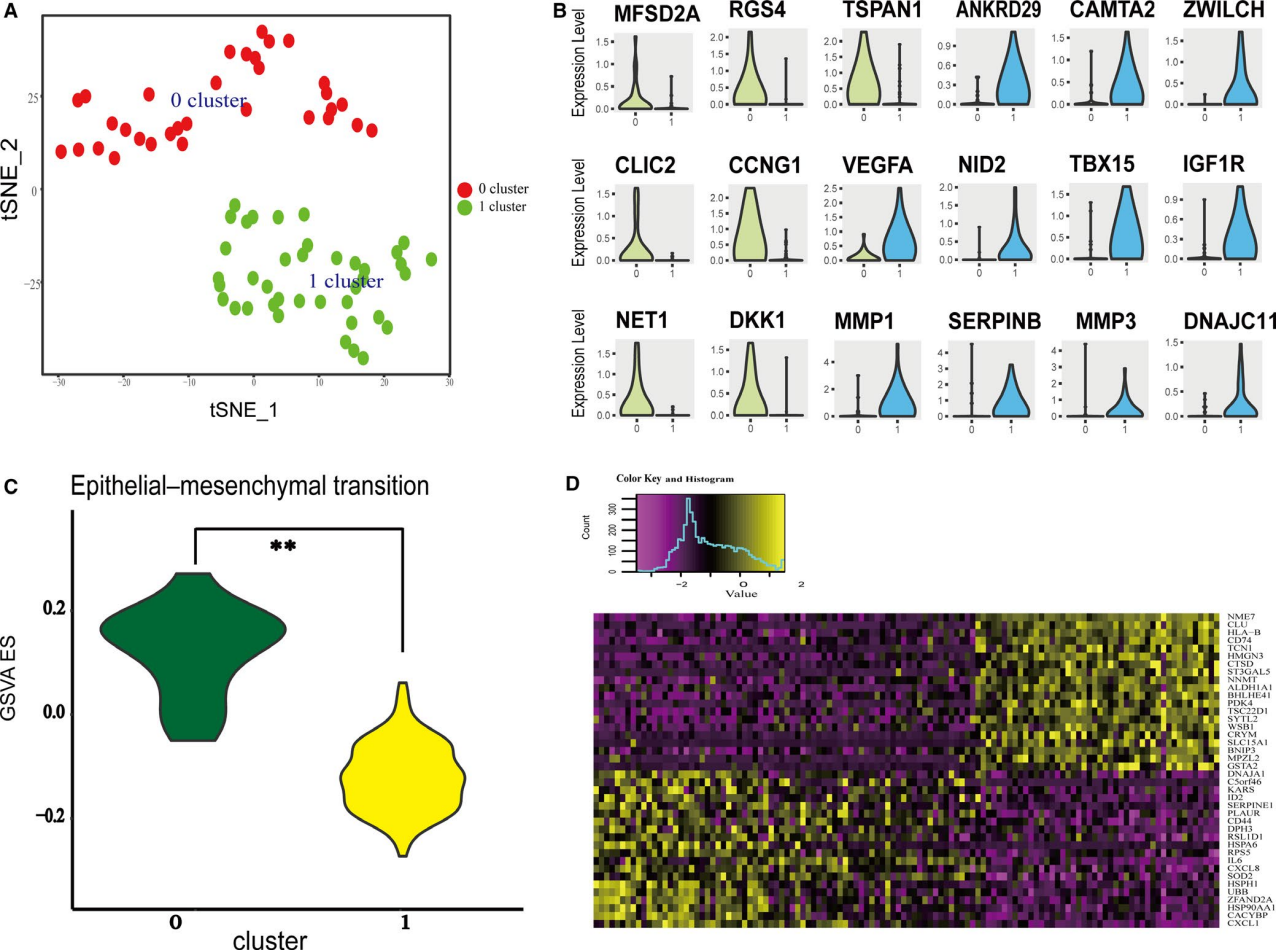
Single‐cell RNA‐seq identified subpopulations from mRCC. A, Cell cluster distribution in mRCC. Two cell clusters were identified and are shown with tSNE maps in cluster 0 and cluster 1 samples, respectively. B, Violin plots of genes showing the highest difference in expression regulation estimates between cluster 0 and cluster 1. C, Violin plots showing the smoothened expression distribution of the cluster‐specific marker genes across the subgroups (GSVA: Gene Set Variation Analysis; ES: enrichment score). D, Heatmap of genes showing the highest difference in expression between cluster 0 and cluster 1

To generate a comprehensive view of cellular diversity in RCC metastases, we selected metastasized cells for further analysis. We applied PCA to HVGs across all 71 metastasized cells (n = 1602 genes) using tSNE on the informative PCs (n = 8). We generated two‐dimensional maps of the data using tSNE and partitioned the cells into two main clusters (Figure 3A). Marker gene expression in cell clusters was dissimilar. Individual clusters were labelled with subpopulation‐specific marker genes and stratified into the EMT pathway‐related cluster (EMT‐RC) (yellow) or the VEGF pathway‐related cluster (VEGF‐RC) (blue) for characterization (Figure 3B). GSVA analysis suggested that subclonal populations of ITH contribute to induction of EMT in metastatic cancer cells (Figure 3C). A heatmap revealed the differential expression patterns of the top 40 DEGs from the two clusters (Figure 3D). In this analysis, EMT‐RC and VEGF‐RC were identified as the main changeable subclonal populations associated with distinct signalling pathway activation in the tumour metastatic samples (Figure S2). The analysis implied considerable heterogeneity within the mRCC cell populations, which indicates that the EMT‐RC and VEGF‐RC subpopulations are major functional subpopulations extensively expanded in mRCC. These differences reflect well‐known disparities in clonal selection following cell evolution in tumour metastasis.^35^

### 3.3 WGCNA and PPI

**FIGURE 4:**
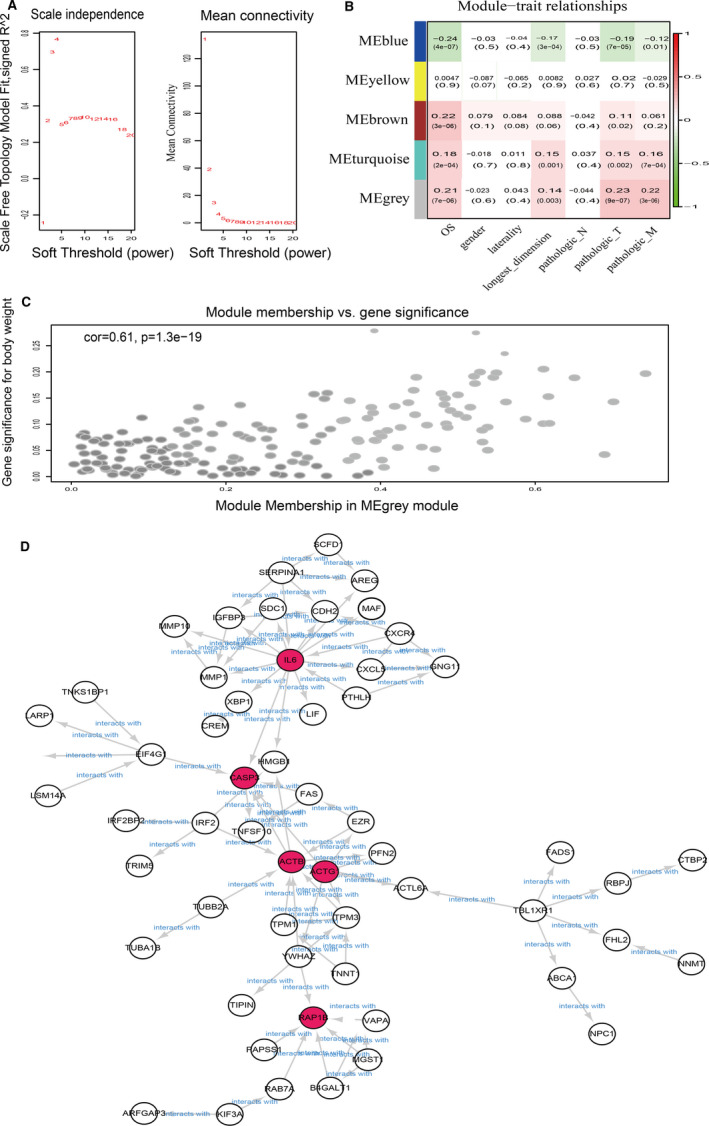
The metastasis‐associated module selection and protein‐protein interaction networks. A, Determination of the soft‐thresholding power in the mRNA WGCNA. B, Module‐trait associations of mRNAs were evaluated by correlations between MEs and clinical traits. Left: Analysis of the scale‐free fit index for various soft‐thresholding powers (β). Right: Analysis of the mean connectivity for various soft‐thresholding powers. C, Scatter plot of the correlation between gene MM in the grey module, which was associated with pathologic metastasis. D, PPI network of DEGs with an interaction score >0.7. Based on STRING and Cytoscape analysis, proteins (the red nodes) with a contribution of greater than six were filtered into the PPI network complex identified as hub genes for the RCC metastatic phenotype, and the red circles were referred to the sub‐dif markers

To estimate the relative contributions of the DEG subpopulations of the two clusters to bulk RNA expression, we applied WGCNA, which is a powerful tool for identifying coexpressing gene modules and relating these modules to external traits, including metastasis. Using a soft threshold power setting of four (Figure 4A), we identified five modules with genes with similar expression patterns in the TCGA‐KIRC RNA‐seq dataset (Figure 4B). The grey module, containing 179 genes, was significantly correlated with metastasis (*R* ^2^ = 0.22, *P* = 3e‐06; Figure 4C). Using the STRING online database and Cytoscape software, a total of 178 coexpressing genes from the grey module were filtered into the PPI network, which contained 87 nodes and 103 edges (Figure 4D). The five most significant nodes in the network were *IL6*, *CASP3*, *ACTB*, *ACTG1* and *RAP1B*, which were identified as hub genes for the RCC metastatic phenotype and are referred to as sub‐dif markers.

### 3.4 Pathway activity analysis of sub‐dif markers

**FIGURE 5:**
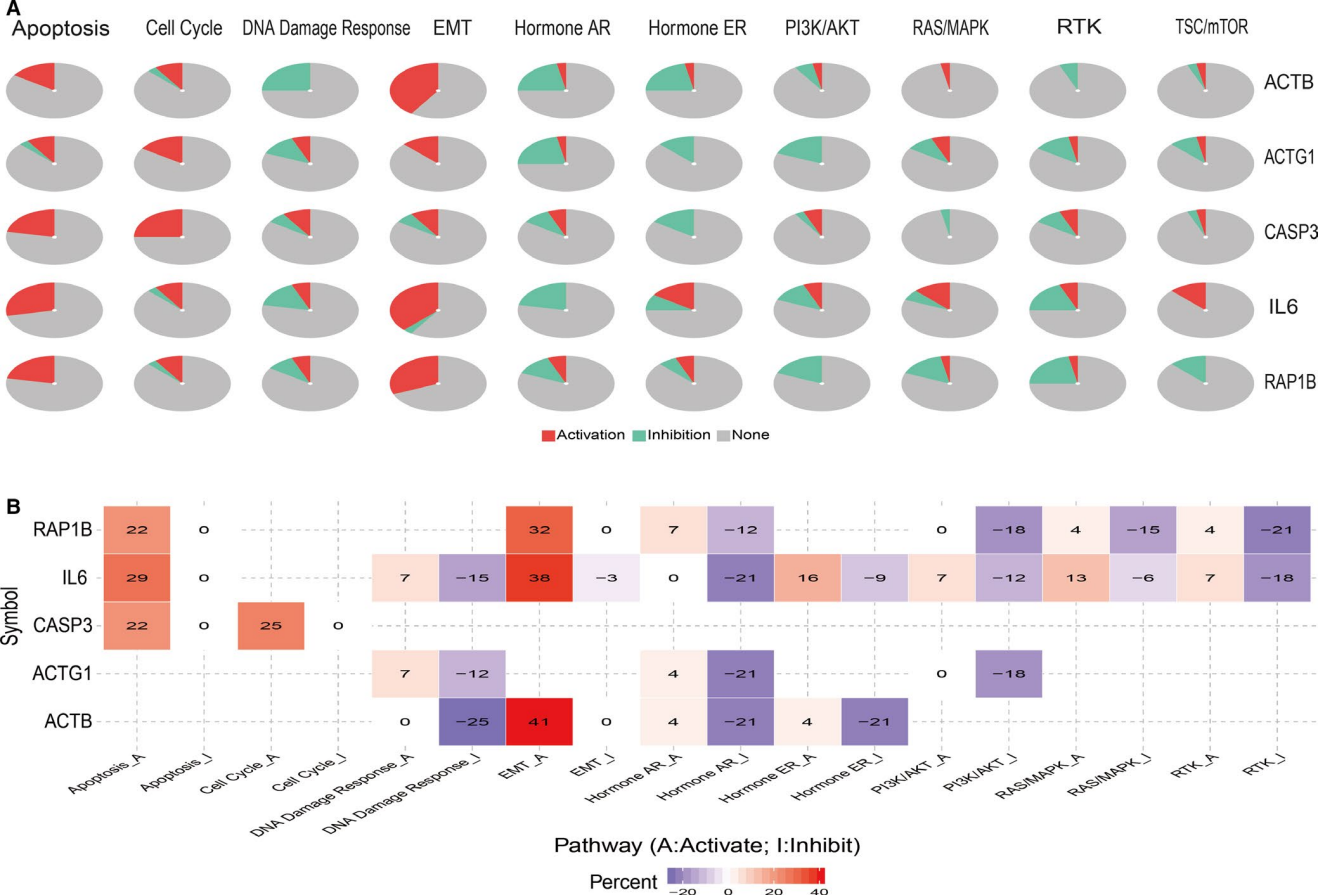
The role of sub‐dif markers in major cancer‐related pathways (GSCALite)

Sub‐dif markers were selected to perform the pathway activity analyses. We identified genes that are widely associated with characteristic metastasis‐related biological processes, such as cell cycle, PI3K/AKT and RAS/MAPK, which are involved in cell proliferation (Figure 5A). The dysregulation of these sub‐dif markers may trigger cell cycle dysfunction and cell proliferation, differentiation and apoptosis pathways, thus inducing tumour cell metastasis, which is in accordance with another independent group's publication.^36^ Some sub‐dif marker genes (*RAP1B*, *IL6* and *ACTB*) were positively correlated with the EMT pathway (Figure 5B). We also identified two positively correlated signalling pathways related to apoptosis and DNA damage response. Thus, we not only confirmed the metastasis‐related pathways frequently reported in other studies, but also verified the strong relationship between sub‐dif markers and the EMT pathway.

### 3.5 Genetic alteration, pathway analysis and drug sensitivity analysis of sub‐dif markers

**FIGURE 6:**
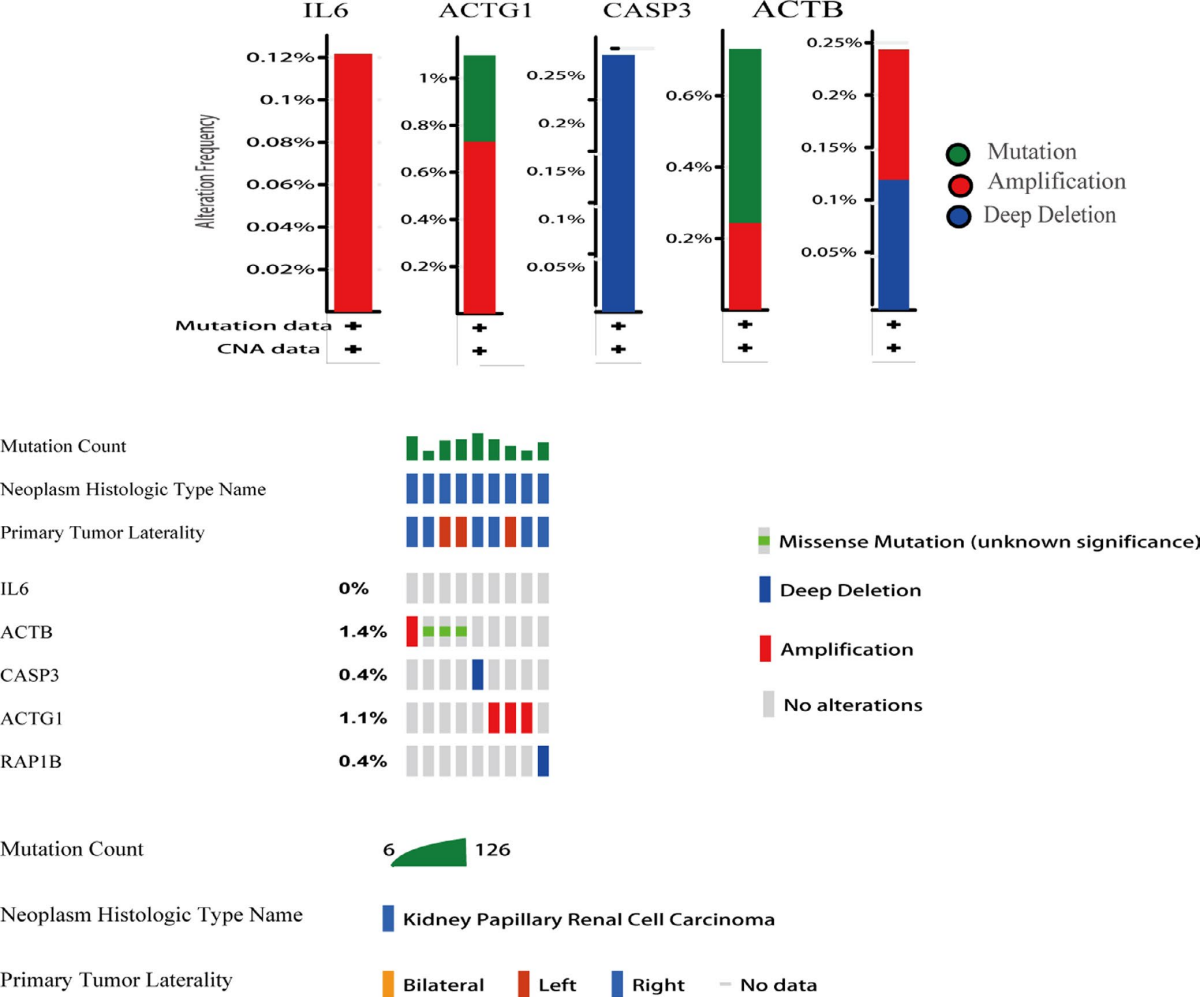
Genetic alterations of sub‐dif markers in RCC determined with the cBioPortal database

**FIGURE 7:**
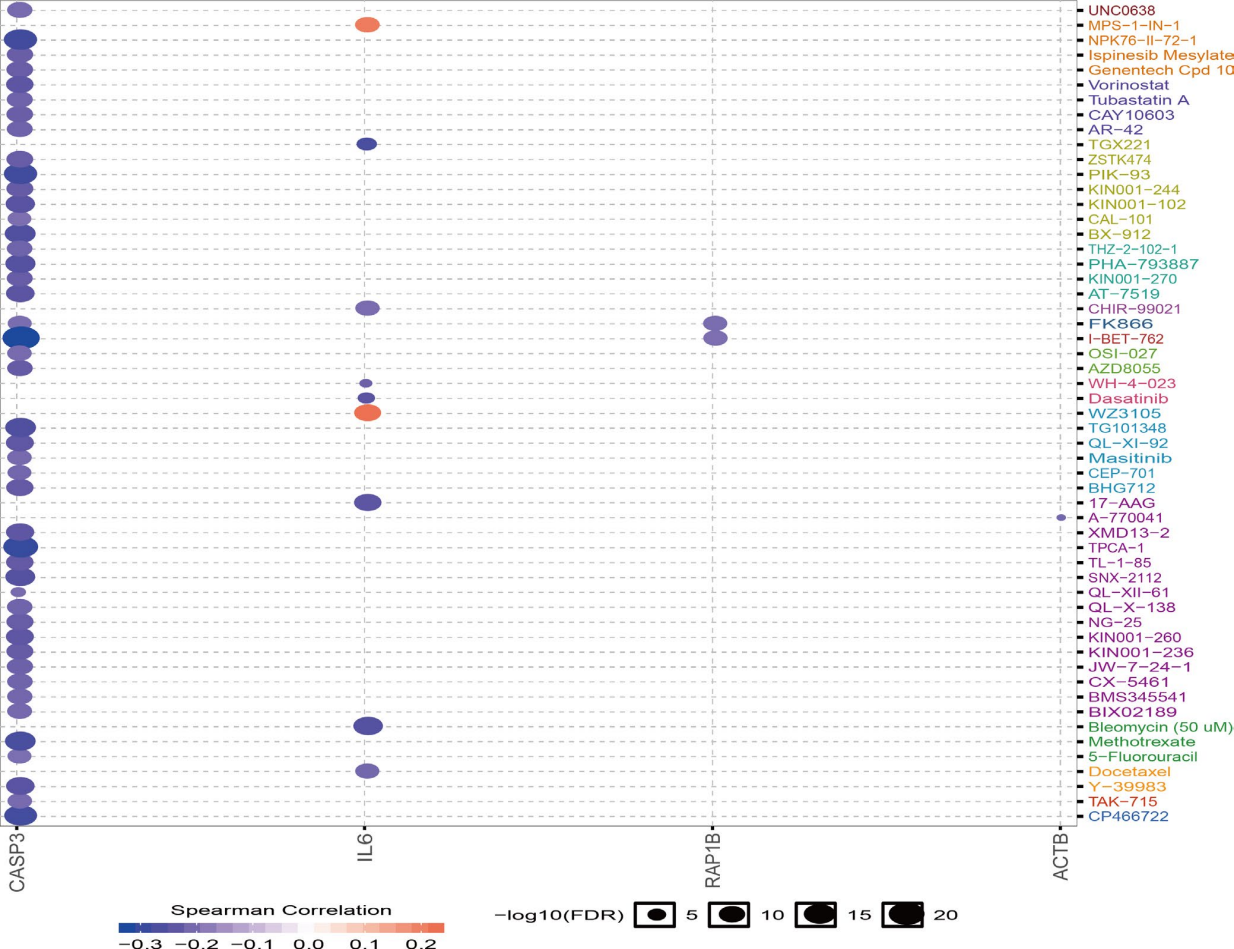
Drug sensitivity analysis of sub‐dif markers in RCC based on the GDSC drug sensitivity database. The Spearman correlation represents the correlation of gene expression with the drug. A positive correlation indicates that high gene expression is resistant to the drug and vice versa

To explore the genetic features of sub‐dif marker expression patterns, we analysed the genetic variation profiles of the sub‐dif markers in 1602 cases that were retrieved from four studies (538 cases from TCGA, Firehose Legacy; 446 cases from TCGA, Nature 2013; 512 cases from TCGA, PanCancer Atlas; and 106 cases from Nat Genet 2013) using the cBioPortal database. We found that the sub‐dif markers exhibited a low mutation frequency in the range of 0.12%‐0.25%. *ACTG1*, *ACTB* and *RAP1B* were well represented with mutation frequencies of 1%, 0.8% and 0.25%, respectively. Specifically, 0.12% of the total patient series harboured only the *IL6* amplification mutation, whereas 0.25% of the patients harboured the *CASP3* deletion mutation (Figure 6). Next, we investigated the effect of sub‐dif markers on drug sensitivity. Using the GDSC database, we found that *IL6* and *CASP3* are potential therapeutic targets. High expression of *CASP3* was correlated with sensitivity to 45 small molecular drugs, while high expression of *IL6* was associated with sensitivity to seven drugs but with resistance to MPS‐1‐IN‐1 and WZ3105 (Figure 7), which may provide further support for the optimization of targeted therapy in patients with mRCC. The nature of the sub‐dif marker response to therapy is unclear, and our data provide a basis for further elucidation of drug sensitivity prediction through these marker genes.

### 3.6 Correlation between sub‐dif marker expression and immune biomarkers and prognosis in KIRC

**FIGURE 8:**
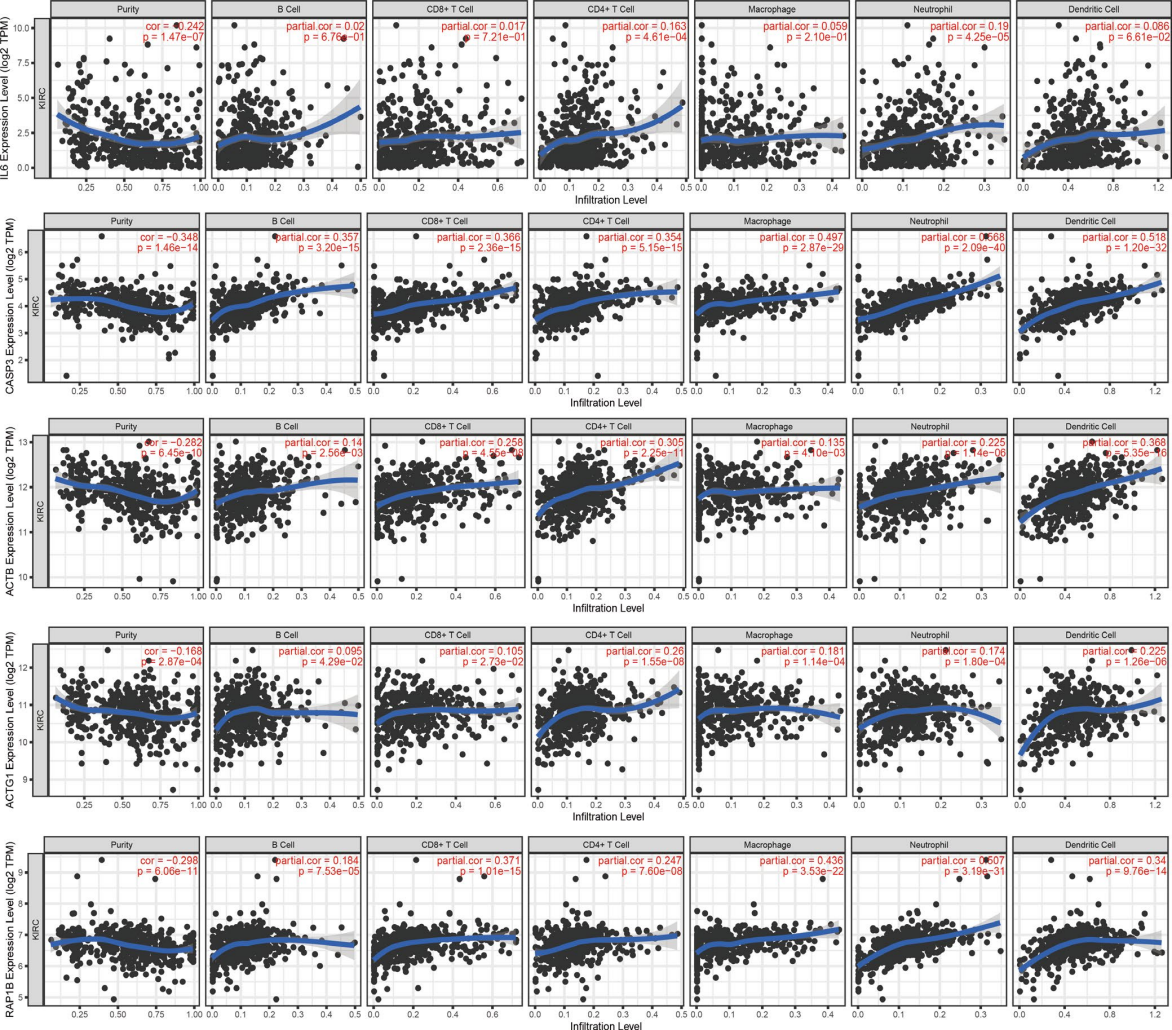
The correlation between sub‐dif marker expression level and the immune infiltration in KIRC (TIMER)

**FIGURE 9:**
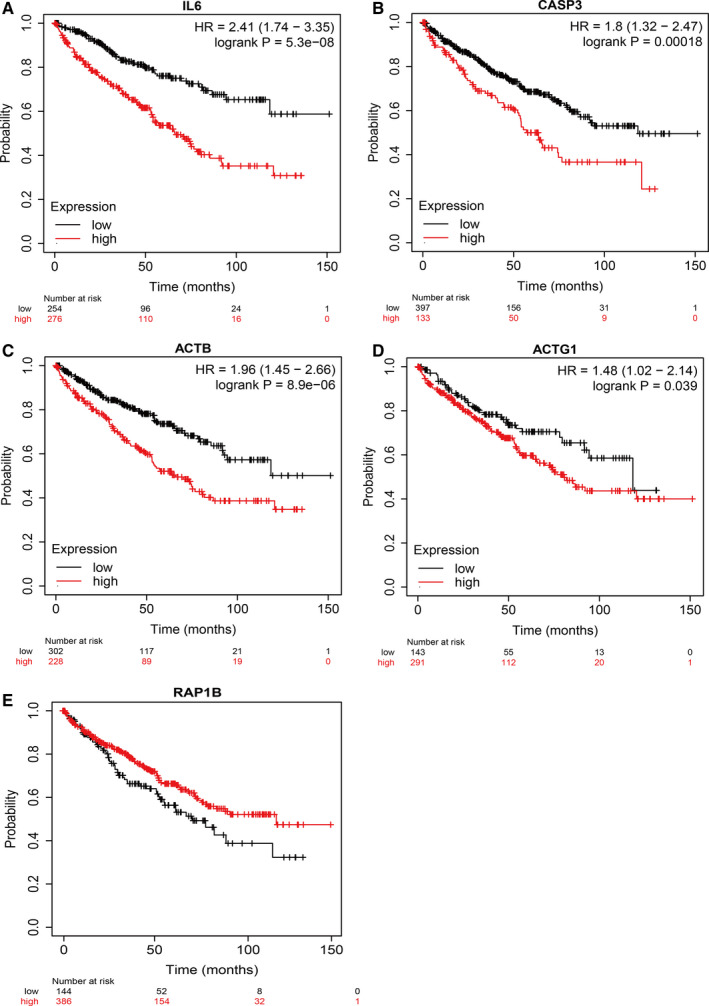
Kaplan‐Meier plot for the survival curve of sub‐dif markers. Kaplan‐Meier survival analysis of tumour samples grouped by gene median expression value c. The sample numbers for each group are shown in brackets. Statistical significance was determined using the log‐rank test (A, IL6; B, CASP3; C, ACTB; D, ACTG1; E, RAP1B)

In the tumour microenvironment, the crosstalk between tumour cells and TILs is essential for tumour metastasis. We further investigated the relationship between the sub‐dif markers and TILs, which is associated with disease prognosis.^37^, ^38^ This analysis showed strong positive correlations between sub‐dif markers and all the TILs, which suggests that the TILs, a hallmark of the tumour microenvironment, are involved in crosstalk with sub‐dif markers and might affect EMT subpopulation differentiation (Figure 8). We also investigated the clinical effects of this population. The prognostic values of the immune cell type fractions and EMT genes were assessed using Cox regression analysis, in which *ACTG1* was identified as a protective factor (HR < 1), whereas the other four genes (*IL6*, *CASP3*, *ACTB* and *RAP1B*) were identified as risk factors (HR > 1) (Table 1). Accordingly, the Kaplan‐Meier plot analysis revealed that higher expression levels of *IL6* (Figure 9A), *CASP3* (Figure 9B), *ACTB* (Figure 9C) and *ACTG1* (Figure 9D) were significantly associated with worse OS. In contrast, higher *RAP1B* (Figure 9E) expression was associated with a better prognosis. These results further validated that sub‐dif markers are of great significance for assessing the prognosis of mRCC.

**TABLE 1:** Cox proportional hazard model analysis

	Coef	HR	95%CI_l	95%CI_u	*P* value	Sig
B_cell	−0.749	0.473	0.025	9.063	0.619	
CD8_Tcell	−1.853	0.157	0.034	0.734	0.019	*
CD4_Tcell	−1.667	0.189	0.013	2.785	0.225	
Macrophage	−1.543	0.214	0.02	2.273	0.201	
Neutrophil	1.463	4.317	0.059	316.55	0.504	
Dendritic	0.685	1.984	0.329	11.971	0.455	
IL6	0.143	1.154	1.063	1.253	0.001	**
ACTB	0.926	2.525	1.47	4.338	0.001	**
CASP3	0.373	1.453	0.91	2.32	0.118	
RAP1B	0.035	1.036	0.661	1.625	0.878	
ACTG1	−0.676	0.509	0.338	0.766	0.001	**

Abbreviations: CI_l, lower confidence interval; CI_u, upper confidence interval; Coef, coefficient; HR, hazard ratio risk.

** *P* < 0.01.

* *P* < 0.05.

### 3.7 Validation of sub‐dif markers by IHC staining

**FIGURE 10:**
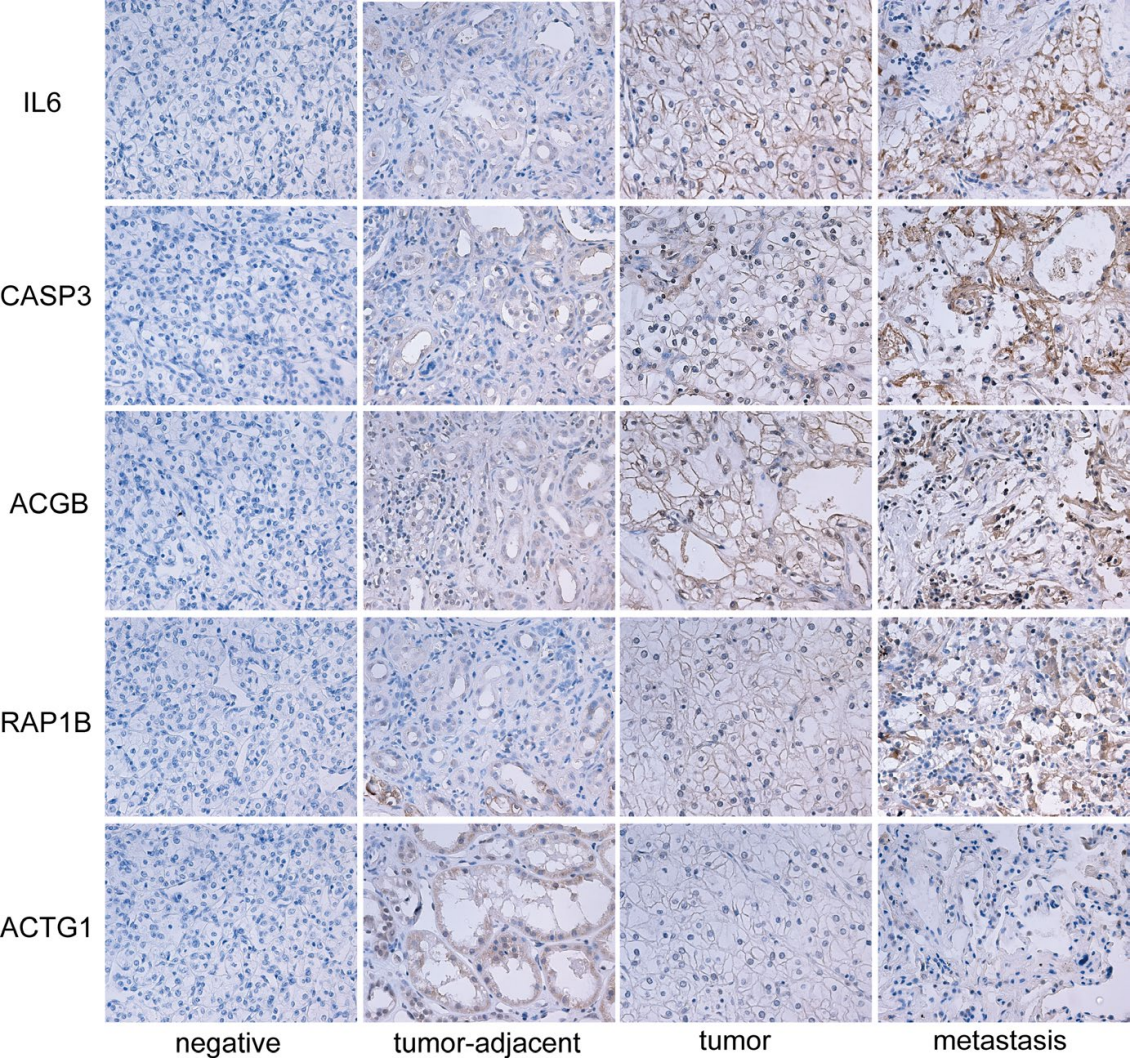
Representative figures showing IHC staining for IL6, CASP3, ACTB and RAP1B and ACTG1. The figures shown in sequence from left to right are negative control, adjacent normal tissues, renal cell carcinoma tissues, lung metastases (×400, for IHC staining)

To support our findings, we sought to provide an independent validation by assessing the protein expression of classifier genes by IHC analysis of primary prostate cancers. We validated the expression and prognostic value of the five candidates, including one protective factor (ACTG1), and four risk factors (IL6, CASP3, ACTB and RAP1B) in RCC patients with lung metastasis (n = 20) by IHC analysis of the bulk RNA‐seq datasets. Medium or high expression of IL6, CASP3, ACTB and RAP1B was observed in 14/20 (~70%) lung metastasis tissues and in 11/20 (~55%) tumour tissues, whereas the expression was negative in the majority of adjacent non‐tumour tissues. In contrast, ACTG1 was stained moderately in 13/20 (~65%) adjacent non‐tumour tissues and showed negative or low expression in the majority of the RCC tissues and lung metastasis tissues (Figure 10). These observations further confirmed our previous results.

## 4 DISCUSSION

In the present study, we used scRNA‐seq to identify two distinct subpopulations in metastatic ccRCC samples. We found evidence of cancer cell heterogeneity within each distinct subpopulation by using different markers. Our findings are analogous to those of other studies, which demonstrated that tumours are transcriptomically heterogeneous.^3^, ^4^, ^39^ The two separate lineages evolve separately into two pathways, EMT‐activated pathways, and VEGF‐related pathways, which apparently include more related genes in the context of metastatic ccRCC. Moreover, we showed that sub‐dif markers from the subpopulations are linked to RCC TILs, drug sensitivity and prognosis. These results indicate that a combined regimen of anti‐vascular therapy and anti‐EMT therapy may be an effective therapeutic strategy for patients with mRCC.

Intratumoural genomic heterogeneity in RCC has been well‐documented.^40^, ^41^, ^42^ Most tumours consist of heterogeneous subpopulations with distinct genotypes, called subclones.^40^ Several studies have indicated that combination therapies targeting multiple subclones may be highly effective against mRCC.^43^, ^44^, ^45^ However, the role of heterogeneity in therapeutic failure and cancer metastasis in metastatic ccRCC remains unclear. Our findings from single‐cell sequencing analysis and the validation of biomarkers in clinical samples support the observations in other studies that identified two cancer metastatic subclones associated with the activation of different pathways. Furthermore, we provided new insights revealing how the heterogeneity of resting metastatic cancer cells predetermines the drug treatment response and prognosis. The two subpopulations identified in our study separately activated EMT‐related and VEGF‐related pathways. GSVA analysis also supported these predictions. Multiple subpopulation‐specific genes were identified. TSPAN1 was found to promote EMT through PI3K/AKT signalling.^46^ Consistent with these results, we identified seven EMT‐related subpopulation‐specific markers (MFSD2A, RGS4, TSPAN1, CLIC2, CCNG1, NET1 and DKK1). Consistent with our observations, the functions of these specifically expressed markers were primarily related to cancer metastasis. CLIC2 is reportedly involved in the formation and/or maintenance of tight junctions, which allows the intravasation of cancer cells.^47^ Cyclin G1 (CCNG1), a target of wild‐type TP53, promotes tumour cell motility by inducing EMT and regulating the Notch3 pathway.^48^ Thus, we described these specifically expressed markers based on their associated pathway activation characteristics, for example, the EMT‐related subpopulation. We also observed the other subpopulation that expressed genes specifically involved in VEGF‐related pathways to promote angiogenesis through IGF‐1/IGF‐1R/ERK signalling.^49^, ^50^ Moreover, we termed VEGF (a key positive regulator of angiogenesis) and some proangiogenic genes (*IGF1*, *MMP1*, *TGFB3*, *PDGFRB* and *PGF*) that were expressed specifically in cluster 1 as the VEGF‐related subpopulation. These observations suggest that each cell plays a different role.^51^ Further exploration of whether the expression of specifically expressed genes can be inhibited to prevent the EMT‐related subpopulation from undergoing a protumorigenic fate or whether the VEGF‐related phenotype can be reverted to improve anti‐angiogenesis efficacy will be valuable.

The single‐cell transcriptome analysis approach has received considerable attention because of repeated attempts to gain an in‐depth understanding of the role of ITH. Herein, we identified potential EMT‐activated subpopulation‐related markers at the gene expression level using transcriptomic data. Accordingly, the EMT programme constitutes the dominant recognized mechanism for initiating the metastatic behaviour of epithelial tumours. Because the roles of EMT in cancer metastasis have been clarified, the biological significance of EMT biomarkers needs to be explored. EMT is a biological process that allows epithelial cells to undergo multiple morphological and biochemical changes, thus enabling them to acquire a mesenchymal cell phenotype, including elevated invasive migratory ability, enhanced resistance to apoptosis, and increased production of extracellular matrix components.^52^

Analysis of the expression profiles of the DEGs between different subpopulations suggested that the sub‐dif markers are important genes that activate EMT‐related pathways to induce metastasis. We identified the two cell populations associated with the activation of different signalling pathways as the most prevalent subpopulations in metastatic cells, and this finding is consistent with those obtained in a study by Elzbieta.^53^ Furthermore, we showed that *IL6*, *CASP3*, *ACTB*, *ACTG1* and *RAP1B* are hub genes that regulate the tumour metastasis regulation network. The expression of these hub genes is related to the prognosis in patients with mRCC. The association between IL‐6 and JAK2, and STAT3 signalling pathway activation and cancer cell migration was observed in a paracrine or autocrine IL‐6‐rich inflammatory environment.^54^ Moreover, IL6 is associated with a network that controls cellular movement, and it serves as an unfavourable prognostic biomarker in terms of overall survival.^55^ Our study revealed that the IL‐6‐rich network hub gene is closely related to the EMT and VEGF subgroups. Using bulk sequencing data from cancerous and normal cell samples, we showed that significant differences in the expression of sub‐dif markers have a high prognostic value, which implies that the sub‐dif markers play crucial roles in the acquisition of the EMT phenotype. This finding is consistent with our hypotheses.

Growing evidence indicates that immune cell infiltration plays an important role of in cancer metastasis, which could affect the prognosis of cancer patients.^56^, ^57^, ^58^ Moreover, immune‐related pathways and immunotherapeutic strategies in cancers are expected to provide a potential direction for cancer therapy.^59^ Notably, the significant associations of sub‐dif markers with TILs, drug sensitivity and OS suggest the potential of using these sub‐dif markers as clinical prognostic biomarkers for predicting the risk in mRCC patients. Some immune biomarkers, such as PD‐1, have been suggested to function as negative immunoregulatory molecules and regulators of cancer cell immune evasion.^60^ Thus, sub‐dif markers may play a vital role in immune escape in the mRCC microenvironment.

In summary, we demonstrated that utilization of ITH in metastatic subpopulations with different pathway activities can facilitate the development of a combined treatment strategy with favourable prognoses. Subgroup heterogeneity leads to the activation of different pathways and therefore provides reasons for selecting targeted combined immunotherapy. Examination of gene expression in single cancer cells not only provides a rationale for combinatorial anti‐VEGF and TKI therapies, particularly PD‐1‐directed therapies, but also paves the way for future investigations on the effects of ITH on primary or acquired resistance to targeted therapy. However, larger cohorts are required to screen other subgroups and aberrantly activated signalling pathways in mRCC.

### 4.1 Limitations

Our study had some limitations. First, we analysed only metastatic tumour samples and could not elucidate the results in the primary tumour samples. Second, we did not compare our findings with those of other independent cohort studies, which is essential for validating our findings and could have yielded more reliable results.

## CONFLICT OF INTEREST

The authors report no conflicts of interest in this work.

## AUTHOR CONTRIBUTION

**Kun Liu:** Data curation (equal); Investigation (equal). **Rui Gao:** Data curation (equal); Formal analysis (equal); Investigation (equal); Methodology (equal). **Hao Wu:** Formal analysis (equal); Investigation (equal); Methodology (equal). **Zhe Wang:** Conceptualization (equal); Funding acquisition (equal); Methodology (equal); Visualization (equal); Writing‐original draft (lead); Writing‐review & editing (equal). **Guang Han:** Conceptualization (lead); Funding acquisition (lead); Project administration (lead).

## Supporting information

Fig S1

Fig S2

## Data Availability

The datasets analysed during the current study are available from the corresponding author on reasonable request.
