# Supplementary figures and images for: Single‐cell analysis reveals metastatic cell heterogeneity in clear cell renal cell carcinoma
Source: J Cell Mol Med. 2021 Mar 23;25(9):4260–74. doi: 10.1111/jcmm.16479 (PMC8093989; doi:10.1111/jcmm.16479)

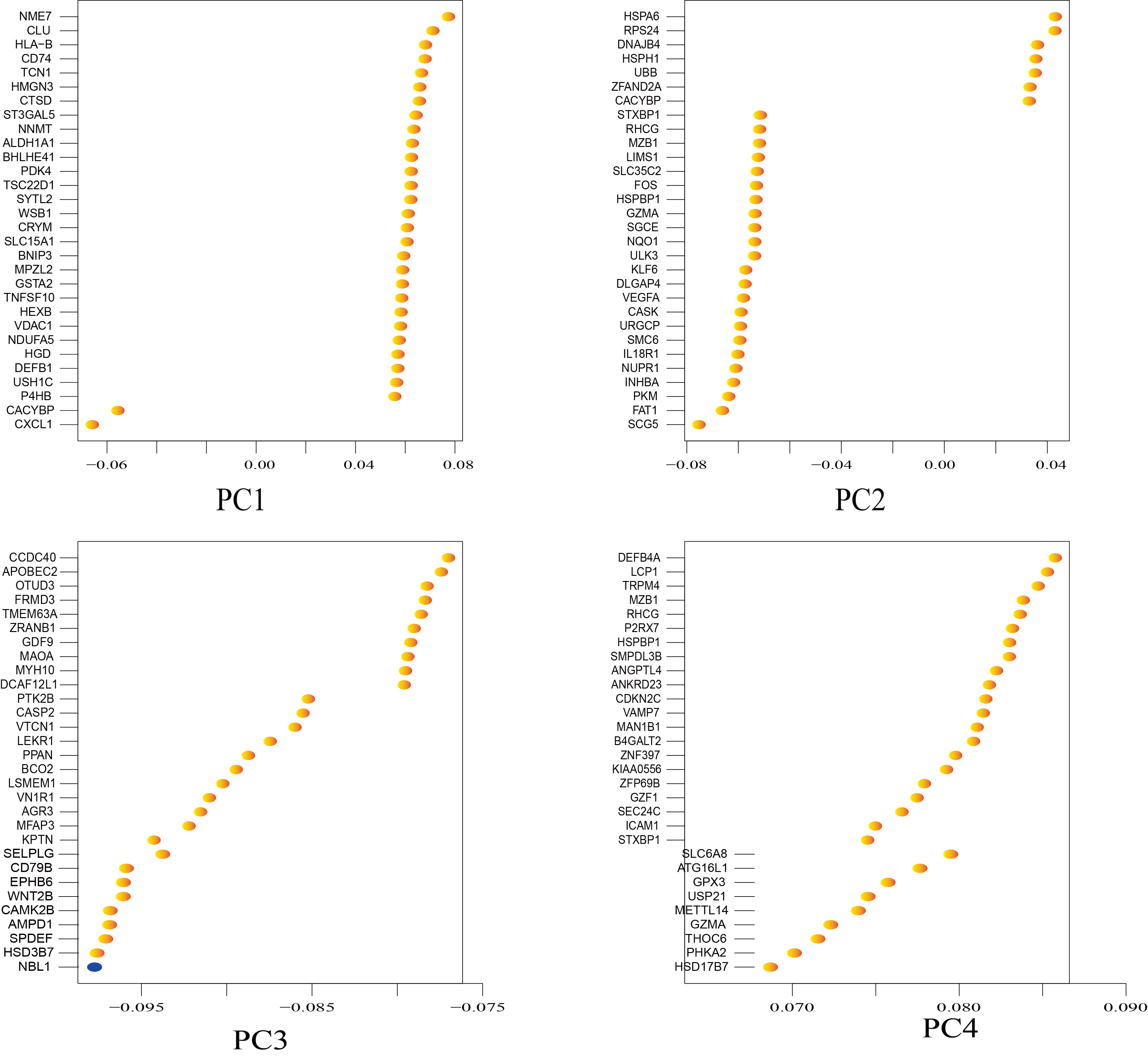

Supplement: Supplementary file 1 — Fig S1 [file JCMM-25-4260-s001.tif]

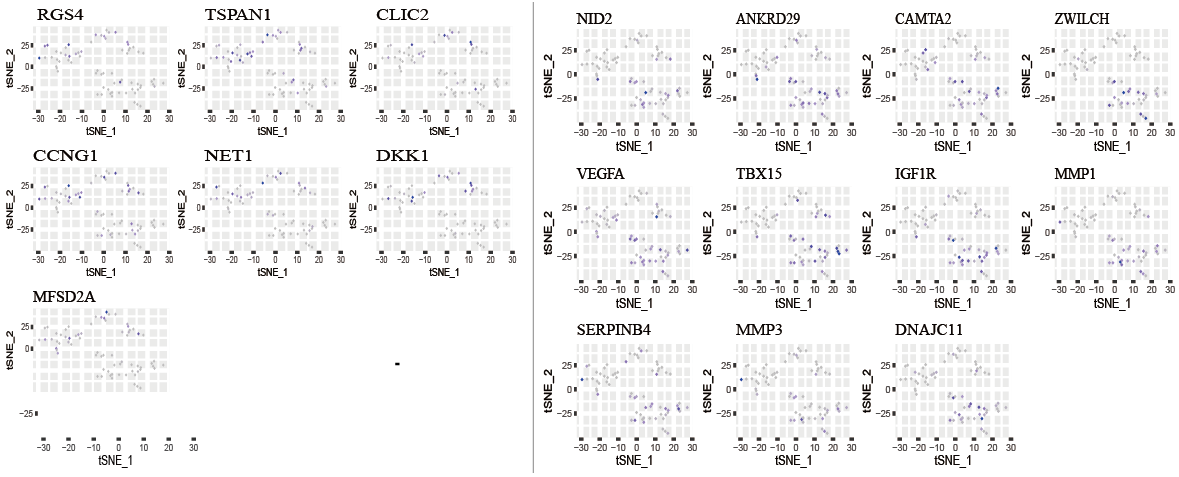

Supplement: Supplementary file 2 — Fig S2 [file JCMM-25-4260-s002.tif]
